# Diffusion model-based inverse design of photonic crystals for customized refraction

**DOI:** 10.1515/nanoph-2025-0499

**Published:** 2025-12-16

**Authors:** Ruotian Lin, Cheng Zhang, Wangqi Mao, Jiahao Ge, Hongxing Dong, Long Zhang

**Affiliations:** Hangzhou Institute for Advanced Study, University of Chinese Academy of Sciences, Hangzhou, 310024, China; Shanghai Institute of Optics and Fine Mechanics, 71182 Chinese Academy of Sciences, Shanghai, 201800, China; University of Chinese Academy of Sciences, Beijing, 100049, China

**Keywords:** photonic crystal, machine-learning, negative refraction, metamaterial

## Abstract

Photonic crystals (PhCs) have demonstrated great potential for use in integrated photonic systems. However, traditional design methods often struggle with low efficiency and limited flexibility. While deep learning approaches offer innovative solutions for the inverse design, existing generative models like generative adversarial network and variational autoencoders still face challenges such as training instability or excessive noise. Here, a novel generative design framework based on the diffusion model is presented to achieve the inverse design of high-precision and customized refraction structures. A comprehensive dataset consisting of operating frequency, refracted angles and corresponding structure patterns is constructed by calculating the equifrequency contours of various PhCs at a resolution of 64 × 64. Based on this dataset, customized PhC structures are successfully generated by using a diffusion model combined with the U-Net model. This design can predict cell patterns with allowable incident and refraction angles ranging from 0°∼80° and −80°∼80°, respectively. And if the types of structures in the dataset are increased, the solution space can be further expanded. A normalized design approach ensures adaptability to multi-scale scenarios. Finite-difference time-domain simulations and numerical analysis indicate that 85 % of the 1000 tested refracted angle errors measured by L2-norm are below 0.1. Such strong correlation between targets and simulated results demonstrates the high stability and precision of our diffusion model-based approach, which may provide a promising avenue for the automated inverse design of photonic devices.

## 1 Introduction

Photonic crystals (PhCs), which are artificially engineered periodic structures capable of manipulating electromagnetic waves through their unique photonic properties, have emerged as important components in modern integrated photonic systems, such as PhC lasers [1], [2], [3], on-chip spectrometers [4], [5], and photonic circuits [6]. Particularly within the silicon photonic platforms, their refractive-index modulation at subwavelength scales generates special refractive effects, facilitating the implementation of high-performance integrated photonic circuits, such as low-loss waveguide [7], [8], [9], [10], nanobeam filters [11], on-chip dispersion control [12], [13], [14], and on-chip beam splitters [15]. Traditional PhC design predominantly relies on experience, where engineers repeatedly combine analytical calculations (e.g., plane-wave expansion methods) with numerical simulations to manually explore parameter spaces [16], [17]. Although this approach has yielded functional designs, it requires significant computational resources when addressing complex multi objective optimization challenges [18]. Recent gradient-based optimization algorithms and genetic algorithms have achieved success in some applications; however, they are affected by the degrees of freedom [19].

Recently, the integration of machine learning techniques has opened new frontiers in the PhC design [20], [21], [22], [23], [24], [25], [26], [27], [28], [29]. Some researchers have employed regression-based neural networks for the highly accurate predicting of photonic properties, such as the photonic bandgap [28], resonant frequency [20], [23], [24], bound states in the continuum [22]. Besides, some have employed generative deep learning approaches, such as generative adversarial networks (GANs) [27], [29] and variational autoencoders (VAEs) [25], for the design of photonic structures with high degrees of freedom. In brief, GANs excel at producing high-freedom, data-like designs via adversarial training, whereas VAEs provide a smooth, interpretable latent space that facilitates controlled exploration and optimization. Diffusion models have recently emerged as a promising technology in generative modeling, demonstrating remarkable success in generating high-fidelity images and videos. Unlike traditional approaches, diffusion models offer training stability while maintaining exceptional resolution capabilities through unique Markov-chain-based denoising processes [30]. These characteristics make diffusion models well-suited for photonic inverse design problems that require both geometric precision and diverse solution exploration.

In this study, we proposed a novel generative design framework that integrates PhC equifrequency contour calculations (EFC) [31], [32], [33] with advanced diffusion modeling to achieve a customized design for specific refracted angles. We calculated the EFC of the PhCs and obtained training datasets based on the EFC theory. Subsequently, a diffusion model combined with a U-Net neural network model is successfully trained. For a given input frequency, incident angle, and refracted angles, the network generates specific refractive designs as 64 × 64-pixel images. Finite-difference time-domain (FDTD) simulations and separate verification using 1000 sets of data were conducted, and the experimental results showed that 85 % of the refracted angle errors were less than 0.1 (L2-norm). Results indicate that our proposed diffusion model-based method has both high stability and high accuracy, paving a new way for the automated inverse design of photonic devices.

## 2 Results and discussion

### 2.1 Design principle of photonic device

**Figure 1:**
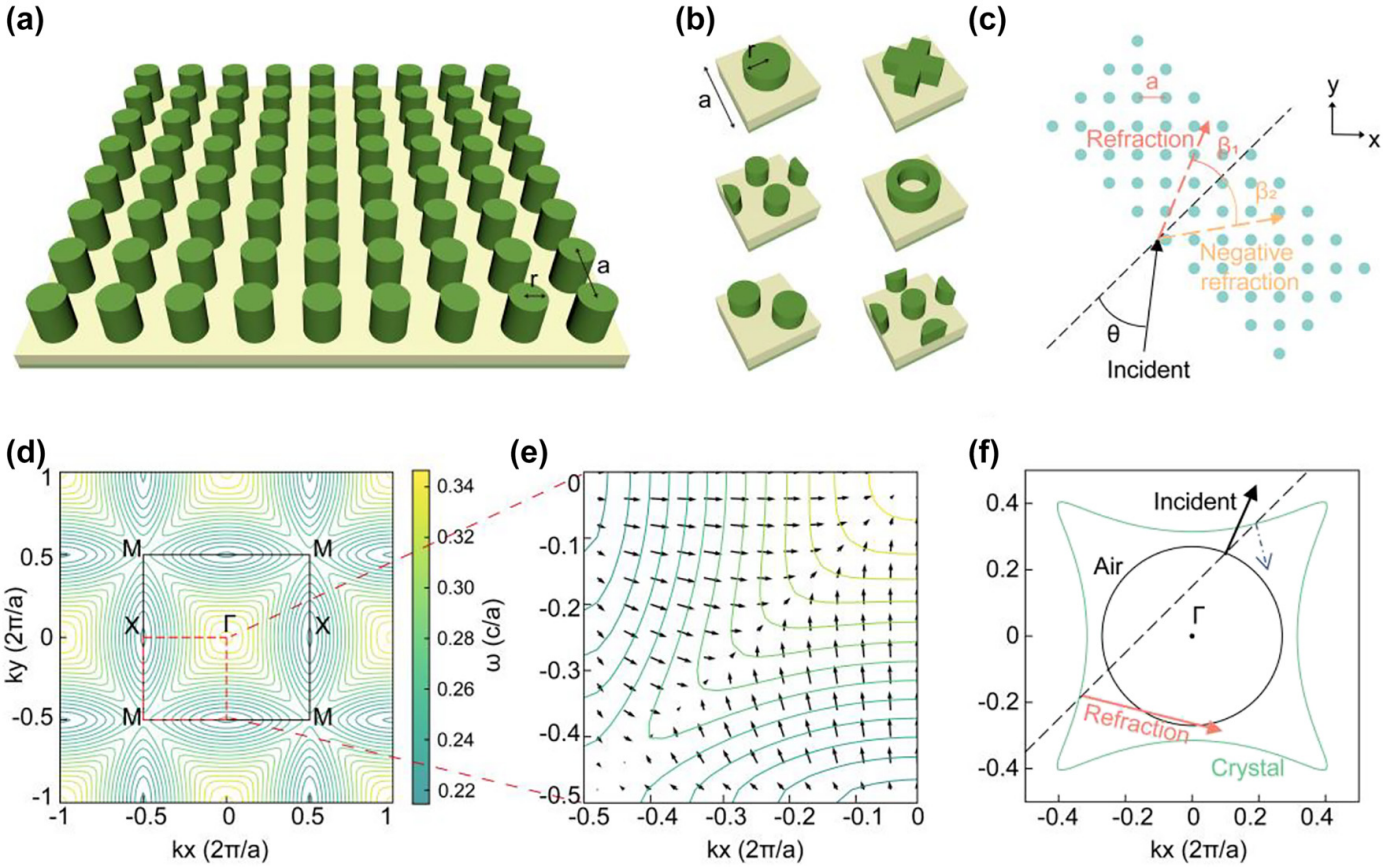
Schematics of structure and principle of a refractive PhC device. (a) PhC slab. (b) Six classic unit cell structures with variable parameters. (c) Sketch map of refraction when light of a specific frequency is incident at a specific angle. (d) EFC of PhC. The black box represents the first Brillouin zone. (e) Gradient direction of EFCs which denotes the group velocity direction. (f) Refractive principle in the EFC theory.

Figures 1a and b illustrate the schematic design of a refractive PhC device. The structure consists of periodically arranged in-plane dielectric rods, incorporating six representative unit cell geometries: circular, X-shaped, sp-kagome, ring, double-lattice, and Lieb (Figure 1b). The unit cells have variable dimensions and create extensive design spaces (detailed in section S1 of the Supplementary Information). Because of the scale-invariant property of Maxwell’s equations, we employed normalized structural parameters to describe the device dimensions.

The refracting device enables precise optical-path manipulation, achieving beam refraction, negative refraction, and birefringence for specific incident light beams (Figure 1c). Light-path modification in PhCs originates from periodic structural modulation, which can be systematically analyzed through EFCs of the photonic band structure. The EFCs represent the frequency curves in k-space (Figure 1d). Following the group velocity formula Vg = ∇ω/k, the gradient direction obtained from the EFC determines the group-velocity direction of the wave vectors, which corresponds to the light propagation directions within the crystal (Figure 1e).

Next, we determine the point in k-space to which the refracted light generated by the incident light corresponds. In Figure 1f, the black arrow indicates the incident direction, and the orange arrow denotes the refracted direction. The blue dashed arrow marks a fake (i.e., physically inadmissible) refracted direction. The green and black contours represent the equifrequency curves of the photonic crystal and air, respectively. An equifrequency curve refers to the set of wave vectors in the energy band at a fixed frequency. In reciprocal space, the full EFC representation comprises the projection of the entire frequency band structure, whereas an equifrequency curve isolates the contour corresponding to a single frequency. Equivalently, it can be viewed as the intersection between a constant-frequency plane and the three-dimensional band surface. The determination of these quantities proceeds as follows.

Step 1: The frequency of light is constant; therefore, we determined a single equifrequency curve (green contour in Figure 1f).

Step 2: Owing to the translational symmetry of the PhCs, the wave vector component of the incident light parallel to the boundary between the two media will remained unchanged. We defined a line perpendicular to the interface (black dashed line in Figure 1f) to represent a constant parallel wave vector. When the vertical line intersects the EFC, the refracted light mode can be determined from the intersection points.

Step 3: In Figure 1f, two refraction direction (orange solid line and blue dashed line) can be seen originating from the intersection point. Because the energy propagation agrees with the group velocity direction, only the outward-pointing gradients from the interface represent physically valid solutions. Only the orange arrow matches.

**Figure 2:**
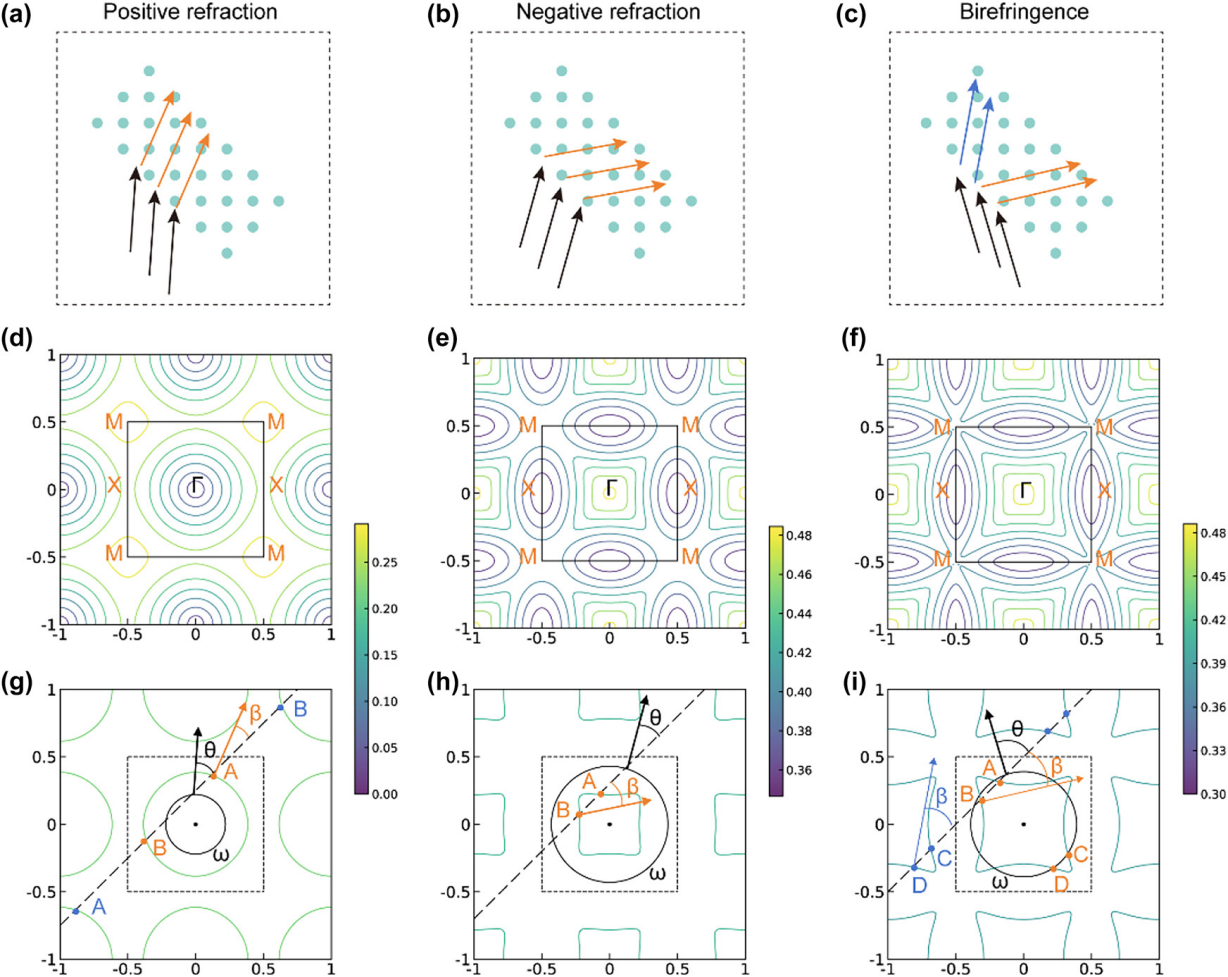
Principles of the positive refraction, negative refraction, and birefringence. (a–c) Refraction diagrams. (d–f) Origin EFC. (g–i) Single equifrequency curve decided by incident light. (g) Orange points A,B and blue points A,B represent the intersections of the vertical line and curve within and outside the FBZ, respectively. The blue points can be equivalently mapped to the corresponding letters in the FBZ. Because of the gradient direction at B is opposite to the propagation direction, the refraction light is determined by A. (h) Intersections outside the FBZ do not appear within the range of the graph, but they are still equivalently mapped into the FBZ. Point B determines a negative refraction. (i) Intersections outside the FBZ are mapped to different points C,D in the FBZ and cause a birefringence.

Figure 2 illustrates the principles of positive refraction, negative refraction, and birefringence in devices. For simplified demonstration, only in-plane incidence on periodic photonic crystals is considered in our work. Notably, non-periodic structures also serve as an effective approach to achieving anomalous beam deflection, such as V-shaped antenna metasurfaces [34], gradient metasurfaces [35], and kirigami-reconfigurable metasurfaces [36]. The mechanisms for generating a single refraction (either positive or negative) have a similar physical foundation. When the vertical line determined by the parallel wave vector component of the incident light intersects with the EFC (Figure 2d–f), intersections may occur both within and outside the first Brillouin zone (FBZ) (dashed box in Figure 2g–i). Owing the periodicity of the crystal, any intersection point outside the FBZ can be equivalently mapped back into the zone through translational symmetry. For single-refraction effects (positive or negative), the folded equivalent point must coincide precisely with the intersection within the FBZ, resulting in a single refracted beam (Figures 2g and h). Conversely, birefringence occurs when the folded equivalent position differs from the original intersection point, thereby enabling the simultaneous generation of two distinct refracted beams (Figure 2i). In summary, the specific refraction phenomenon is determined by the interplay between the wave vector of the incident light and EFC of the PhC structure.

### 2.2 Deep learning procedure by using diffusion and U-net

**Figure 3:**
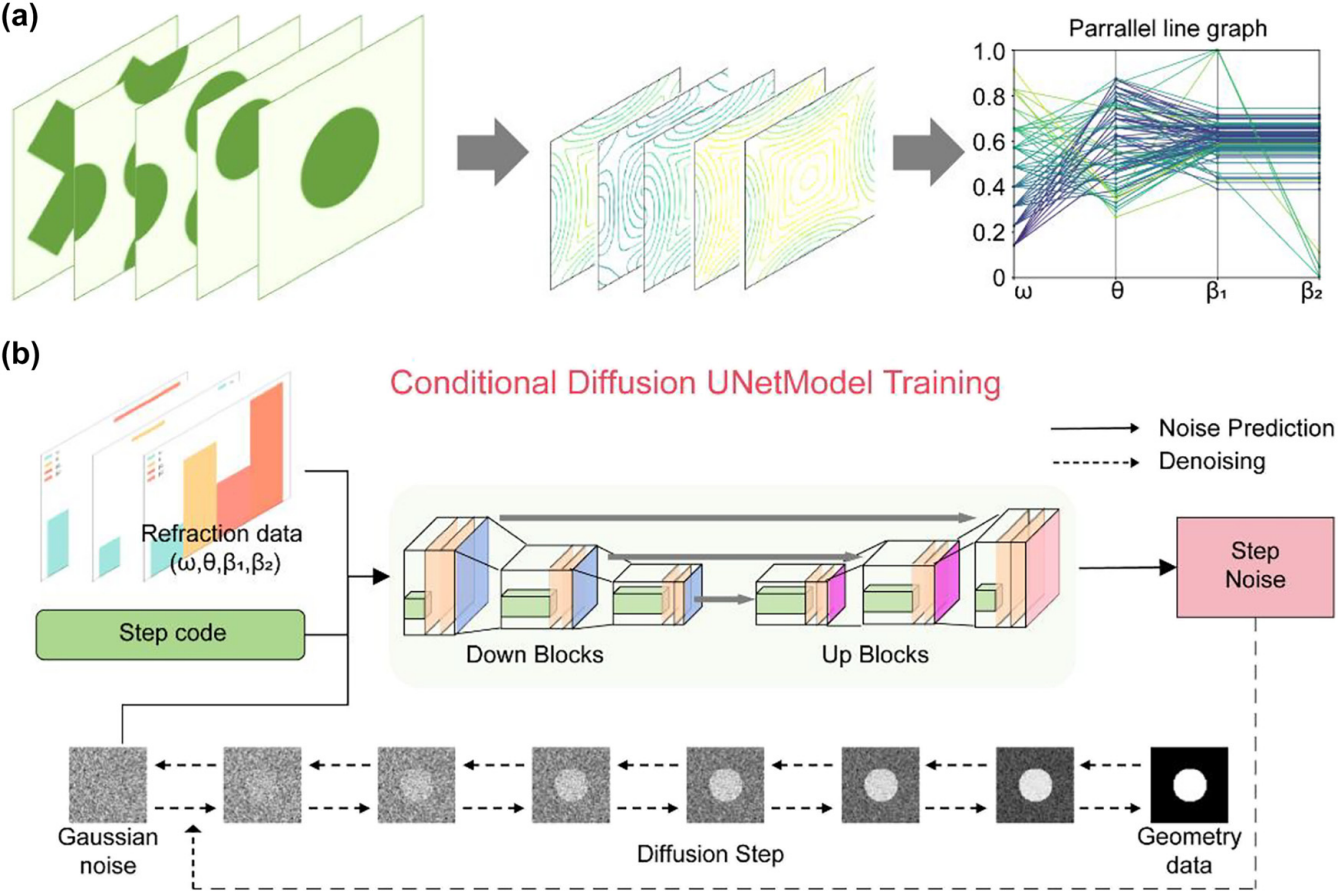
Deep learning process of data preparation and train. (a) Data preparation through the EFC theory. The parallel line graph represents the distribution of a part of data. (b) Conditional diffusion U-net model. The gray part represents the diffusion step. The colorful part represents the noise prediction step.

Designing a structural inverse design process using deep learning fundamentally involves approximating the data distribution between photonic configurations and their optical responses. To establish this mapping, we systematically collected datasets containing photonic unit cell geometries paired with the corresponding refraction parameters, including incident frequency *ω*, angle *θ*, refracted angle *β*_1_, and refracted angle *β*_2_ (when birefringence occurs). For dimensional consistency in machine learning processing, we standardized all refraction outputs to a dual-angle format; when a single refraction occurred, *β*_2_ was intentionally set equal to *β*_1_. This approach maintains uniform data dimensions while preserving the physical significance across both single-refraction and birefringence cases. Notably, these relationships exhibit a nonunique correspondence; a single structure permits multiple incident conditions, whereas identical refraction phenomena may arise from different geometries. Some examples can be found in S2 of the Supplementary Information. The training data collection process is illustrated in Figure 3a. First, we encoded unit cell patterns as 64 × 64 grayscale images, where dark pixels represent high-index materials (*n* = 3) and light pixels denote low-index regions (*n* = 1). Then, we used this method to create 2160 distinct unit cell figures (360 size-varying samples per pattern type). Through an EFC analysis, we computed 10,000 training instances by evaluating random combinations of incidence angles and frequencies. The datasets were partitioned, with 90 % allocated to neural network training and 10 % reserved for performance validation.

We trained a diffusion-based neural network using the collected datasets. Unlike traditional direct-image generation models such as GANs and VAEs, diffusion architectures perform step-by-step denoising processes (Figure 3b). Previous generative inverse design frameworks typically consist of two collaborative neural network components, discriminator-generator pairs in GANs and encoder-decoder structures in VAEs. Inspired by nonequilibrium thermodynamics, diffusion models define a Markov chain of diffusion steps that gradually add Gaussian noise to image samples, and then learn the reverse denoising process to reconstruct target images from the noise (Figure 3b). This method has been proven to be a reasonable and stable generation method. The specific process of this method is as follows. Let *t* represent the diffusion steps, and *x*_*t*_ denote the image at step t. This process can be mathematically described as,

$$x_{t}=\sqrt{\alpha_{t}}x_{t-1}+\sqrt{1-\alpha_{t}}\varepsilon_{t-1}$$

where, *α*_*t*_ represents the weight coefficient at the diffusion step *t*, and *ε*_*t*_ denotes the noise sampled from a standard normal distribution. Following this forward noising process, we implemented a reverse denoising procedure that can be mathematically formulated using Bayes’ theorem,

$$q\left(x_{t-1}|x_{t}\right)=\frac{q\left(x_{t}|x_{t-1}\right)q\left(x_{t-1}\right)}{q\left(x_{t}\right)}$$

The original data of the image can be calculated by combining deep learning to predict the noise *ε*_*t*_ during the denoising process (Figure 3b). Gaussian noise was added to 64 × 64 target geometry maps. This process was then repeated 1,000 times, degrading the structural information to pure noise levels. After that, we trained a neural network to reconstruct the target geometry from these noise-corrupted inputs. In parallel, we condition the model on the refractive information associated with each target geometry to enable controllable, customized outputs.

We used a U-Net neural network to predict noise. A U-Net is a symmetrical neural network structure with a shape resembling a “U’, which is good at processing image data. The left side of the network is downsampling, and the right side is upsampling. Convolution and pooling operations are included in the downsampling process to effectively capture the image information. During the upsampling process shown on the right, a skip connection operation is introduced to fuse the feature information from the downsampling and preserve fine spatial details. We incorporated a condition mechanism to achieve customized refracted angles. By incorporating the refractive information (incident frequency *ω*, angle *θ*, refracted angle *β*_1_ and *β*_2_) corresponding to the PhC structure into the learning process through cross attention, the final generated image was correlated with the refractive information.

### 2.3 Model evaluation by FDTD simulation and numerical analysis

**Figure 4:**
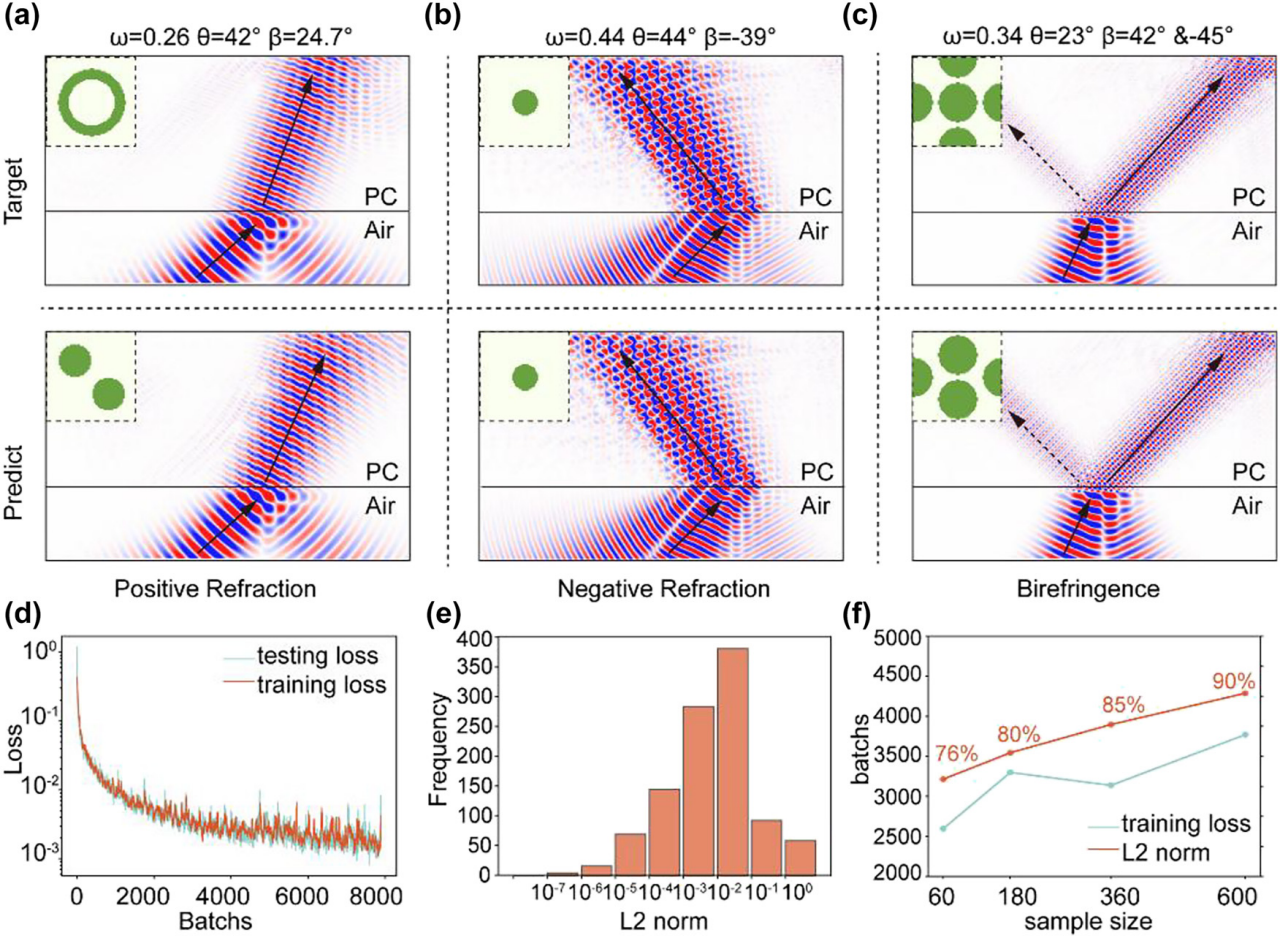
Evaluation result. (a–c) First row: Target simulation images of three kinds of refraction situations and their origin structures. Second row: Simulation images corresponding to the model-suggested structures when target refraction data have been input. (d) Training and testing loss. (e) Distribution histogram of L2-norm for 1000 sets of validation data. (f) The training results vary with the number of samples.

Figure 4e illustrates the training results through the loss curve, where the training loss decreases progressively with batches, demonstrating stable convergence. To evaluate model performance, we conducted comprehensive testing using held-out test datasets. Three target structures (“Target” row of Figure 4) were selected to cover three kinds of responses: positive refraction, negative refraction and birefringence. These structures were subsequently analysed through FDTD simulations using the open-source software Meep. The simulations implemented single frequency Gaussian beam sources, with light incident at specified angles into PhC slabs, successfully reproducing the calculated refractive phenomena (“Target” row of Figure 4) by the EFC theory. When provided with target refraction parameters, our inverse design model generated candidate structures (“Predict” row of Figure 4). The FDTD results qualitatively confirmed the agreement between the predicted and actual optical behaviour across all three refractive responses. Notably, a comparative analysis of the target and designed structures (Figure 4a and c) revealed that different configurations can achieve identical refraction effects, consistent with the nonunique structure – function relationship in the datasets. For a quantitatively assessment, we randomly sampled 1,000 test cases and computed the refracted angles via an EFC analysis under the same frequency and incident angle conditions. Performance was quantified using the L2-norm,

$$\text{L}2\text{Norm}=\sum {\left(\beta_{i}-{\hat{\beta }}_{i}\right)}^{2}$$

A quantitative error analysis was performed using the L2-norm to evaluate the deviations between the true results and inverse-designed predictions. As shown in Figure 4e, 85 % of the generated structures exhibited errors below 0.1, demonstrating the capability of the model to reliably produce configurations that satisfy the target design.

We compared results obtained from four training-set sizes. For each structural class, we sampled 60, 180, 360, or 600 structures and then fixed the total number of refraction examples at 10,000 via calculation. We also drew a common validation set of 1,000 randomly selected examples. After independently training a model on each dataset, we tracked the training loss over optimization batches (blue line in Figure 4f) and measured the proportion of validation samples with an L2-norm error below 0.1 (orange line in Figure 4f). More detail in Section S3 of the Supplementary Information. We found that the number of optimization batches required to reach a training loss of 0.015 increased with dataset size, whereas validation accuracy increased monotonically. The former indicates slower convergence on larger datasets, as the model must accommodate greater variability and learn a smoother denoising mapping rather than memorizing data. The latter reflects improved generalization: additional samples provide richer conditional signals, yielding better alignment between conditioning and generated structures. Balancing sample size and validation accuracy, we selected the model trained with 360 samples per class as our research subject.

**Figure 5:**
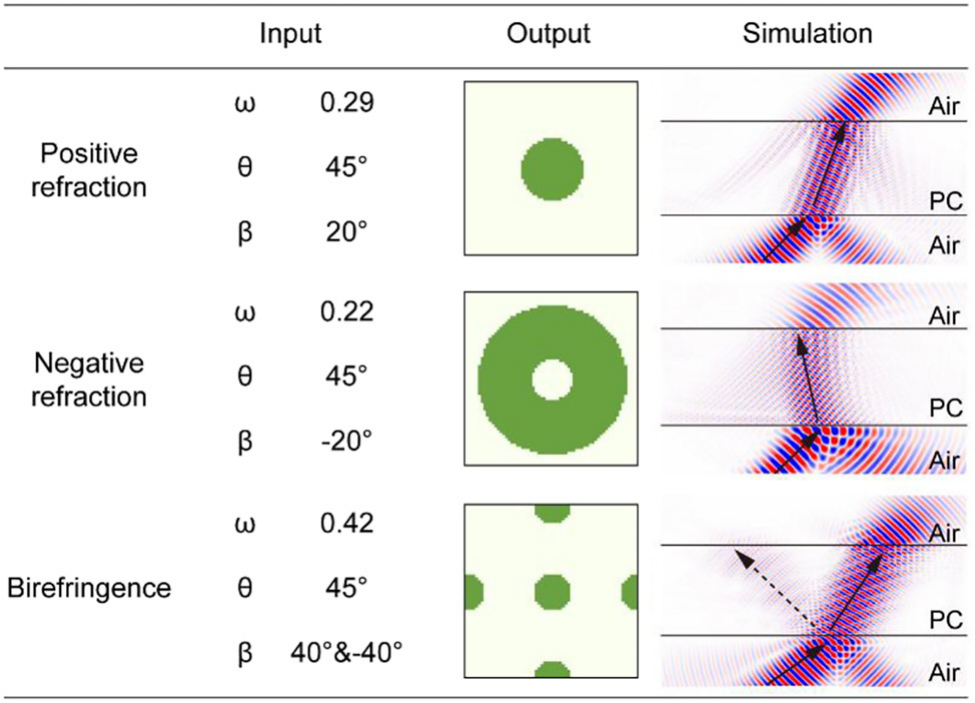
Suggestion results of manually input data.

In addition, we used manually designed parameters for target design, which represent three types of refraction situations: (1) *ω* = 0.29, *θ* = 45°, *β* = 20° (2) *ω* = 0.22, *θ* = 45°, *β* = −20° and (3) *ω* = 0.42, *θ* = 45°, *β1* = 40°, *β2* = −40°. The generated structure and simulation results are shown in Figure 5. The results show good consistency, indicating that the model can provide an ideal reference design solution.

## 3 Conclusions

In summary, we presented a diffusion-based deep learning framework for the inverse design of custom refraction structures. Using the EFC theory, we calculated the angles and frequency information. We then constructed a correspondence between the refractive information and photonic structure, including single positive refraction, single negative refraction, and birefringence configurations. This study presents a design framework operates effectively with incident angles from 0°∼80° and refraction angles from −80°∼80°, and its normalized nature ensures applicability across multi-scale scenarios. Finally, verification through FDTD simulations showed that the model achieved highly accurate results; 85 % of the 1000 tested refracted angle errors (measured by the L2-norm) were below 0.1. This indicates that our method has high stability and accuracy and is a feasible approach for achieving automated photonic design systems.

## 4 Methods

### 4.1 Diffusion model

We adopt a denoising diffusion probabilistic model with 1,000 training timesteps and a squared-cosine beta schedule (squaredcos_cap_v2). The model is trained to predict Gaussian noise (epsilon prediction) using a mean squared error (MSE) objective. Our backbone is a conditional UNet2D with input resolution 64 × 64. The network takes 5 input channels (1 image channel concatenated with 4 conditioning channels: w, *θ*, β1, β2 broadcast to spatial maps) and outputs 1 channel. We use 2 residual layers per block, channel widths [64, 64, 128, 256], downsampling blocks [Res, Attn, Attn, Attn] (i.e., DownBlock2D, AttnDownBlock2D × 3), and upsampling blocks [Attn, Attn, Attn, Res] (i.e., AttnUpBlock2D × 3, UpBlock2D). Training is performed with the Adam optimizer (learning rate 0.001), batch size 256, for 20 epochs.

### 4.2 Finite-difference time-domain

We perform finite-difference time-domain simulations in Meep using a Gaussian-beam source. A photonic-crystal slab is placed at the center of the domain, and the beam is launched from air at a specified incident angle. The spatial resolution is set to 10, and the total simulation time steps to 300.

## Supplementary Material

Supplementary Material Details
