# Supplementary material for: Diffusion model-based inverse design of photonic crystals for customized refraction
Source: Nanophotonics. 2025 Dec 16;14(27):5537–44. doi: 10.1515/nanoph-2025-0499 (PMC12717900; doi:10.1515/nanoph-2025-0499)
Supplement: Supplementary file 1 — Supplementary Material Details [file j_nanoph-2025-0499_suppl_001.docx]

Supplementary Information

Diffusion model-based inverse design of photonic crystals for customized refraction

**Ruotian Lin^123^, Cheng Zhang^123^, Wangqi Mao^23^, Jiahao Ge^123^, Hongxing Dong^123^, Long Zhang^23^**

**^1^ Hangzhou Institute for Advanced Study, University of Chinese Academy of Sciences, Hangzhou 310024, China.**

**^2^ Shanghai Institute of Optics and Fine Mechanics, Chinese Academy of Sciences, Shanghai 201800, China.**

**^3^ University of Chinese Academy of Sciences, Beijing 100049, China.**

**Supplementary Section S1：Selection of Six Structures**

Based on the previously reported literature about the photonic crystals, six unit-cell structures were deliberately selected. Regarding the circular structure, it was the most famous and simplest pattern in photonic crystal research, while the X-shaped structure as a deformation of the circular one, showcased the specified C4 symmetry. In contrast to the abovementioned solid constructions, ring-shaped structure can be regarded as the complementary type. For the double-lattice structure, Sp-kagome structure, and Lieb structure, multi lattice structures were contained within one unit cell to generate various electromagnetic responses as well as improve the dimension and richness of the database.

Normalized lattice constant here was adopted to express the structural parameters of the selected six types of microstructures. The side length of the tetragonal lattice was set to 1. Other structure parameter information and the specific scope is as follows:

1. Circular structure: Radius from 1/16 to 15/16;
2. X-shaped structure: The X-shape is composed of two identical rectangles, that one of them is rotated 90 degrees. Rectangular short axis from 1/16 to 5/16 and long axis from the length short axis of to 15/16;
3. Ring-shaped structure: Radius from 1/8 to 15/16. Radius from 1/16 to the length of large radius;
4. Double-lattice structure: Radius from 1/16 to 15/16;
5. Sp-kagome structure: Radius from 1/16 to 1/4;
6. Lieb structure: Radius from 1/16 to 1/4.

**Supplementary Section S2：Different structures with the same refractive phenomenon**

Figures S1, S2 illustrate the three-dimension energy band of the structures which can more intuitively display the difference between structures. As in Figs. S1, S2, achieving identical refraction effect with different configurations will not cause the same equifrequency contours that are the contour map of the energy bands. Meanwhile, incident frequencies have been changed to show the different refraction (one is birefringence while another bandgap) in Fig S1, as well as incidence angles (one is birefringence while another single refraction) in Fig S2.


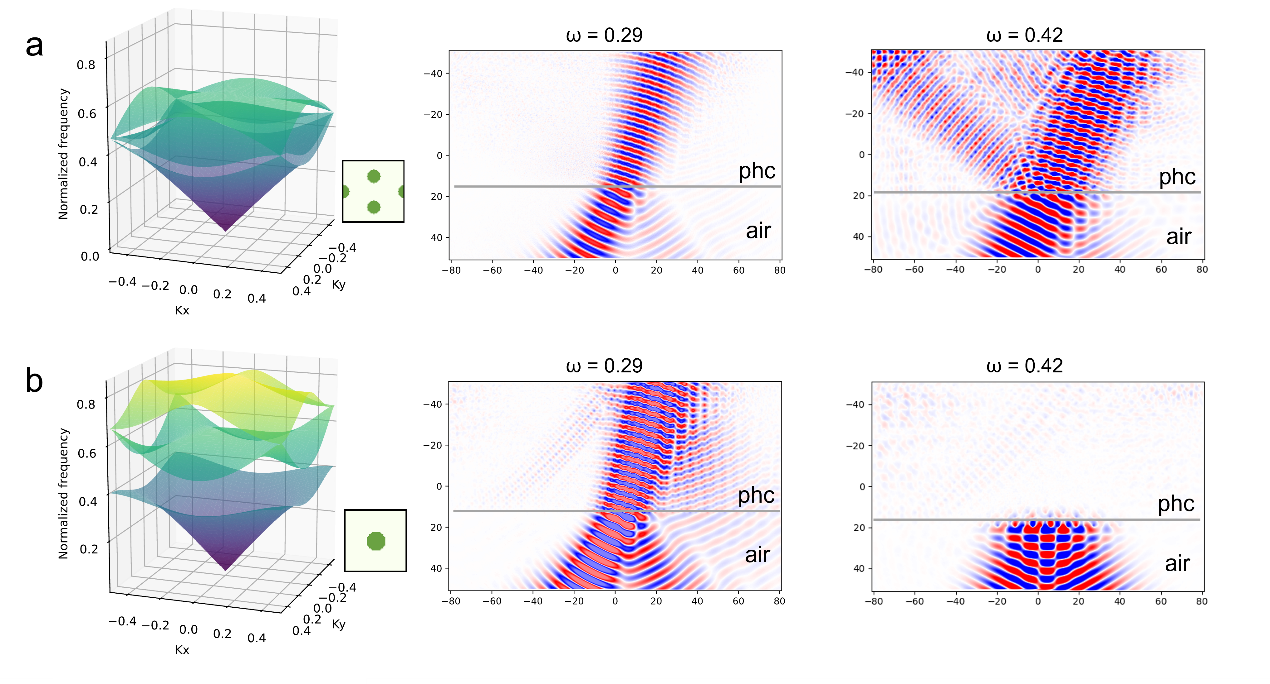


**Figure S1.** Energy band diagram of the structure in Fig.4(a). And refraction between different frequencies.


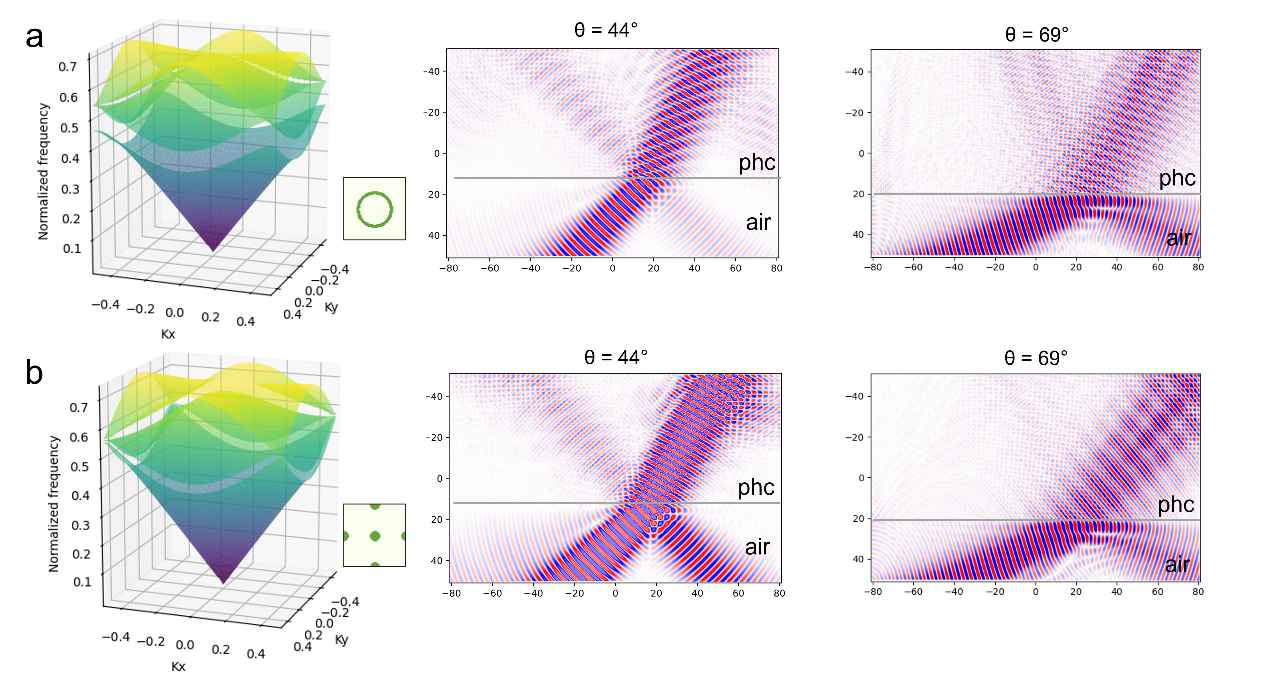


**Figure S2.** Energy band diagram of the structure in Fig.4(c). And refraction between different incidence angles.

**Supplementary Section S3： Training results on different sampling datasets**


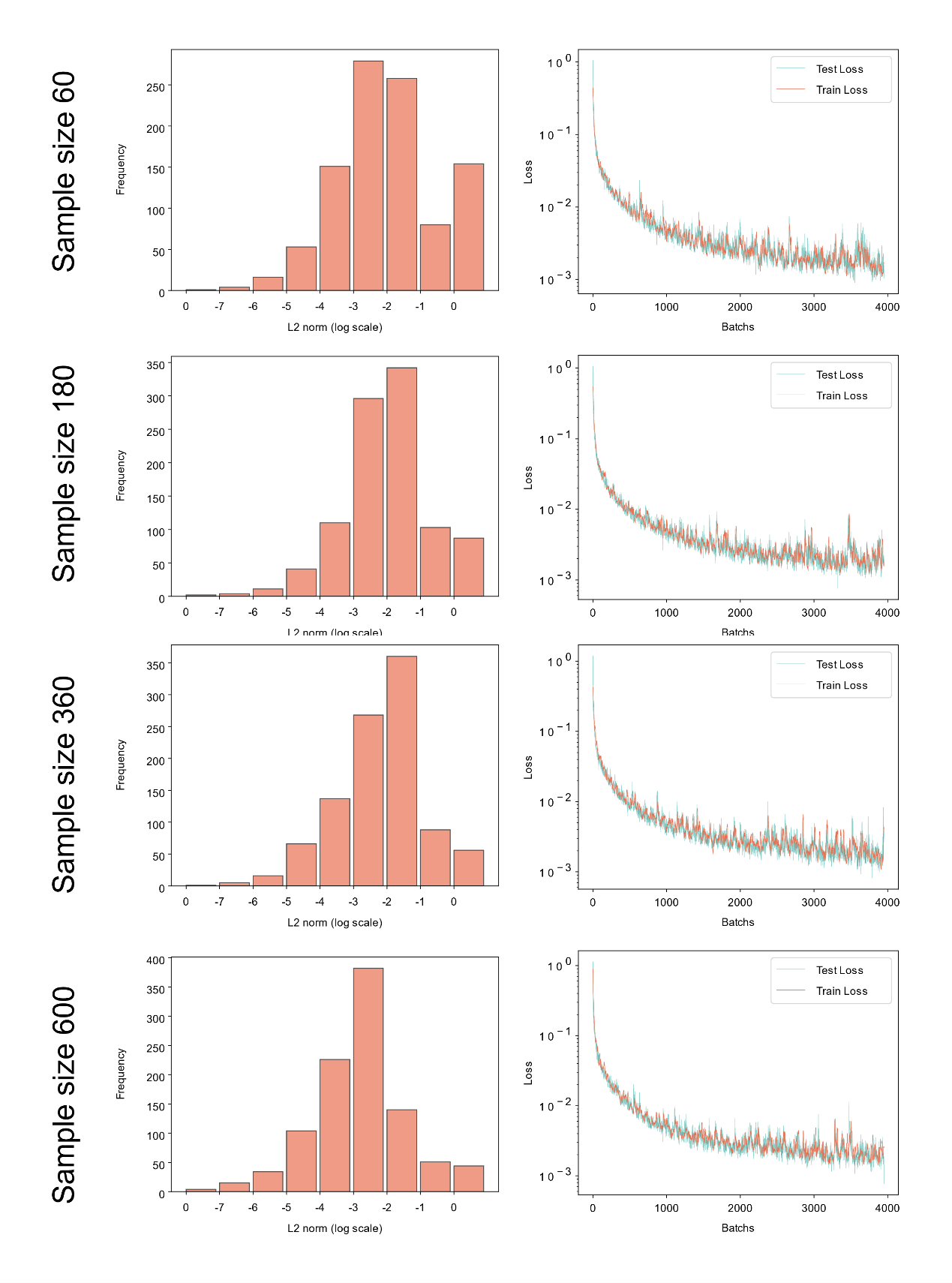


**Figure S3.** Four datasets with different sampling sizes, training loss and l2-norm validation for individual training.
